# Suppression of Pax3–MITF-M Axis Protects from UVB-Induced Skin Pigmentation by Tetrahydroquinoline Carboxamide

**DOI:** 10.3390/ijms21249631

**Published:** 2020-12-17

**Authors:** Yong-Pyo Choi, Ga Hyun Kim, Song-Hee Kim, Jongseo Maeng, Heesoon Lee, Sang-Bae Han, Ki Ho Kim, Youngsoo Kim

**Affiliations:** 1 College of Pharmacy, Chungbuk National University, Cheongju 28160, Korea; na_v_er_hood@naver.com (Y.-P.C.); kimsole10@naver.com (G.H.K.); songhee.kim1@gmail.com (S.-H.K.); maengjongseo19940521@gmail.com (J.M.); medchem@chungbuk.ac.kr (H.L.); shan@chungbuk.ac.kr (S.-B.H.); 2 Kihobio Company, Cheongju 28160, Korea; kihobio1@hanmail.net

**Keywords:** tetrahydroquinoline carboxamide, melanin pigmentation, tyrosinase, microphthalmia-associated transcription factor isoform M, paired box gene 3, guinea pig skin

## Abstract

Paired box gene 3 (Pax3) and cAMP responsive element-binding protein (CREB) directly interact with the *cis*-acting elements on the promoter of microphthalmia-associated transcription factor isoform M (MITF-M) for transcriptional activation in the melanogenic process. Tyrosinase (Tyro) is a target gene of MITF-M, and functions as a key enzyme in melanin biosynthesis. Tetrahydroquinoline carboxamide (THQC) was previously screened as an antimelanogenic candidate. In the current study, we evaluated the antimelanogenic activity of THQC in vivo and elucidated a possible mechanism. Topical treatment with THQC mitigated ultraviolet B (UVB)-induced skin pigmentation in guinea pig with decreased messenger RNA (mRNA) and protein levels of melanogenic genes such as *MITF-M* and *Tyro*. Moreover, THQC inhibited cAMP-induced melanin production in α-melanocyte-stimulating hormone (α-MSH)- or histamine-activated B16-F0 cells, in which it suppressed the expression of the *MITF-M* gene at the promoter level. As a mechanism, THQC normalized the protein levels of Pax3, a transcriptional activator of the *MITF-M* gene, in UVB-exposed and pigmented skin, as well as in α-MSH-activated B16-F0 culture. However, THQC did not affect UVB- or α-MSH-induced phosphorylation (activation) of CREB. The results suggest that suppression of the Pax3–MITF-M axis might be a potential strategy in the treatment of skin pigmentary disorders that are at high risk under UVB radiation.

## 1. Introduction

Skin hyperpigmentation (pigmented spot) is characterized by an excess increase in the number of melanocytes, melanosome biogenesis with melanin pigmentation in melanocytes, and melanosome transfer to keratinocytes [1]. Solar ultraviolet B (UVB) radiation is a major factor governing acquired pigmentation in the skin [2,3]. Melanogenic hormones are produced and secreted from epidermal keratinocytes, where UVB radiation stimulates the expression of the pro-opiomelanocortin (*POMC*) gene [4,5]. Following enzymatic processing of POMC polypeptide, α-melanocyte-stimulating hormone (α-MSH) is produced in keratinocytes [6]. In turn, α-MSH binds to melanocortin 1 receptor (MC1R) on the membrane surface of melanocytes in the epidermal/dermal border of skin, which mediates signaling cascade for melanosome biogenesis with melanin pigmentation [7,8]. Microphthalmia-associated transcription factor isoform M (MITF-M) plays an important role in the melanogenic process, as well as in the development and survival of melanocytes [9,10]. Black-brownish eumelanin and yellow-reddish pheomelanin are produced in the melanosome, an organelle of melanocytes [10,11]. Tyrosinase (Tyro) catalyzes a common step, l-Tyr to dopaquinone, in the biosynthesis of both melanins. Eumelanin and pheomelanin pathways diverge from dopaquinone [12]. Tyro-related protein 1 and dopachrome tautomerase (DCT) are indispensable to produce eumelanin pigment [12]. On the other hand, pheomelanin pigment is derived from conjugation by Cys or glutathione [11]. Melanosomes are then delivered to keratinocytes in the overlaying epidermis for skin pigmentation [13].

Several MITF isoforms are expressed from the distinct promoters of the *MITF* gene in tissue-specific manners [14]. Among these, melanocyte-specific MITF-M is inducible in response to UVB radiation or α-MSH/MC1R signaling for facultative melanogenesis [15,16]. A number of *cis*-acting elements on the MITF-M promoter are positively or negatively regulated by transcription factors that are associated with signaling pathways in the melanogenic process [17]. Paired box gene 3 (Pax3) interacts with the *cis*-acting element at −260 to −244 on the MITF-M promoter, which upregulates the expression of the *MITF-M* gene in a synergistic manner with the SRY-related HMG-box (SOX10) [18,19]. The cAMP responsive element-binding protein (CREB) through cAMP signaling, ubiquitous in almost all cell types, is adapted to drive the melanocyte-specific transcriptional activation of MITF-M via tight cooperation with SOX10 [20]. The *cis*-acting elements on MITF-M promoter responding to CREB or SOX10 are located at −147 to −140 or at −282 to −261, respectively [19]. In melanocyte development by Wingless-type (Wnt) signaling, β-catenin is stabilized and accumulated in the cytoplasm, and then allowed to translocate into the nucleus, where it stimulates the promoter activity of the *MITF-M* gene through coactivation of the lymphoid enhancer-binding factor 1 (LEF1) [21]. Interestingly, Pax3 inactivates the DCT promoter in the absence of Wnt signaling, in which Pax3 forms a transcriptional repressor complex with LEF1 and displaces MITF-M [18].

**Figure 1:**
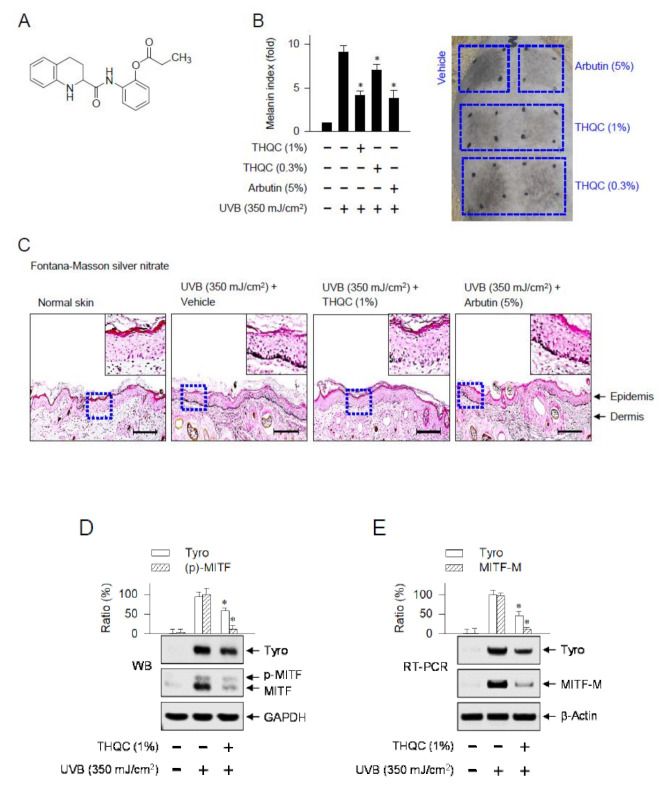
Effect of tetrahydroquinoline carboxamide (THQC) on skin pigmentation in guinea pig. (**A**) Chemical structure of THQC. (**B**–**E**) Dorsal skin of guinea pig was irradiated with ultraviolet B (UVB) and treated topically with THQC according to the protocol in Figure S1 (Supplementary Materials). (**B**) The melanin index was measured in UVB-exposed area of skin and is represented as relative fold. A photograph of pigmented skin is also presented. The vehicle was a mixture of propylene glycol, ethanol, and water (5:3:2). (**C**) Skin tissue was sectioned and then reacted with Fontana–Masson silver nitrate to stain melanin granule as black. (**D**) Protein extract from skin tissue was resolved on SDS acrylamide gel by electrophoresis and subjected to Western blot analysis (WB) with anti-tyrosinase (Tyro), anti-microphthalmia-associated transcription factor (MITF), or anti-glyceraldehyde 3-phosphate dehydrogenase (GAPDH) antibody. (**E**) Total RNA from skin tissue was subjected to semi-quantitative RT-PCR analysis of Tyro or MITF-M with the internal control β-actin and resolved on agarose gel by electrophoresis. * *p* < 0.05 vs. UVB alone.

Melanocyte-specific MITF-M is a transcription factor with the basic helix–loop–helix Leu zipper, and regulates the promoter activity of numerous melanogenic genes, including enzymes in melanin biosynthesis such as Tyro, structural proteins for melanosome biogenesis, and even membrane receptors such as MC1R [9,22]. In the current study, tetrahydroquinoline carboxamide (THQC, Figure 1A) mitigated UVB-induced facultative pigmentation in the dorsal skin of guinea pig, as well as α-MSH-induced melanin production in B16-F0 culture. Moreover, THQC suppressed the expression of the *MITF-M* gene at the promoter level in the skin or B16-F0 culture via decreasing the protein levels of Pax3. The results suggest suppression of the Pax3–MITF-M axis as a potential strategy in the treatment of skin pigmentary disorders that are at high risk under UVB radiation.

## 2. Results

### 2.1. Topical Treatment with THQC Mitigated UVB-Induced Skin Pigmentation

We first carried out UVB-induced skin pigmentation to understand the antimelanogenic activity of THQC in vivo. After shaving, dorsal skins of guinea pigs were topically treated with THQC or arbutin and exposed to UVB light according to the protocol in Figure S1 (Supplementary Materials). UVB-exposed dorsal skins of guinea pigs markedly increased melanin index over the normal skin (Figure 1B). Topical treatment with THQC significantly decreased UVB-induced melanin index, as well as visual pigmentation in the skin, as did arbutin (Figure 1B). There were differences in the decrease of melanin index between 0.3% and 1% THQC (Figure 1B). However, UVB radiation for acquired pigmentation or topical treatment with THQC did not cause any corrosion in the skin (Figure 1B). Arbutin, a Korean Food and Drug Administration (FDA)-approved skin whitener, inhibits the catalytic activity of Tyr in a competitive mechanism [23]. Transdermal absorption of numerous chemicals across the skin of guinea pig displays similar kinetic parameters to the human skin; thus, guinea pig skin can serve as a surrogate [24]. Next, dorsal skins of guinea pigs were biopsied and then serially sectioned. Melanin granules in the skin were stained with Fontana–Masson silver nitrate (Figure 1C). Topical treatment with THQC on UVB-irradiated skin decreased the levels of melanin granules, which was more significant in the basal layer of the epidermis (Figure 1C). Melanocytes at the border between the epidermis and dermis produce melanin pigments in the organelle of melanosome [8].

Furthermore, protein extracts or total RNAs were prepared from skin tissue of guinea pig and then subjected to Western blot analysis or semi-quantitative RT-PCR. Topical treatment with THQC decreased UVB-induced protein levels of Tyro or MITF in the skin, in which anti-MITF antibody recognized the phosphorylated and unphosphorylated forms of MITF (Figure 1D). Accordingly, UVB-exposed skin increased the messenger RNA (mRNA) levels of Tyro or MITF-M, which was also counteracted by topical treatment with THQC (Figure 1E). However, THQC absorbed very little UVB light at 290–320 nm (Figure S2, Supplementary Materials), thus excluding the direct effect of THQC on photoprotection. Ensulizole is a Korean FDA-approved sunscreen agent and was employed as a positive control for UVB absorption [25]. The results suggest that THQC could mitigate the acquired pigmentation in UVB-exposed dorsal skin of guinea pig via suppressing the expression of melanogenic genes such as *Tyro* and *MITF-M*.

### 2.2. THQC Inhibited Melanin Production in cAMP-Elevated B16-F0 Culture

**Figure 2:**
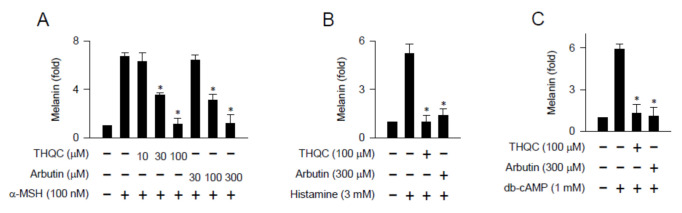
Effect of THQC on melanin production in B16-F0 culture. B16-F0 cells were stimulated with α-melanocyte-stimulating hormone (α-MSH) (**A**), histamine (**B**), or dibutyryl (db)-cAMP (**C**) for 72 h in the presence of THQC. Melanin pigment was quantified by measuring absorbance value at 405 nm and is represented as relative fold. * *p* < 0.05 vs. α-MSH, histamine, or db-cAMP alone.

We examined whether THQC could affect melanin production in B16-F0 culture since it inhibited the generation of pigmented melanosome in the epidermis/dermis border of UVB-exposed skin (Figure 1C). Melanogenic hormones including α-MSH are secreted from keratinocytes [4]. α-MSH binds to its specific receptor MC1R on the membrane surface of melanocytes, which stimulates cellular signaling for melanin production [7]. B16-F0 cells markedly increased the intracellular and extracellular levels of melanin pigments upon exposure to α-MSH alone (Figure 2A), even though the use of the B16 melanoma cell line but not normal human melanocytes has a limitation. Treatment with THQC dose-dependently inhibited α-MSH-induced melanin production in the cells, as did arbutin (Figure 2A). THQC was more effective, about fivefold, than arbutin in view of the half maximal inhibitory concentration (IC_50_) value (Figure 2A). Histamine is also secreted from the mast cells and keratinocytes in UVB-exposed skin and, thus, can generate inflammatory conditions such as erythema and stimulate melanin production in delayed kinetics [26,27]. Histamine has melanogenic activity, generating the pigmented melanin granules in epidermal melanocytes, through its specific binding to the H_2_ receptor [27]. Upon exposure to histamine alone, B16-F0 cells enhanced the levels of melanin pigments (Figure 2B). Treatment with THQC or arbutin inhibited histamine-induced melanin production in the cells (Figure 2B).

We then employed dibutyryl (db)-cAMP as a cell-permeable cAMP agonist, since G-protein-coupled receptors such as MC1R and H_2_ stimulate the melanogenic process via cAMP as a second messenger [7,27]. Upon exposure to db-cAMP alone, B16-F0 cells markedly increased melanin pigmentation over the basal condition (Figure 2C). Treatment with THQC or arbutin inhibited db-cAMP-induced melanin production in the cells (Figure 2C). However, THQC did not affect the viability of B16-F0 cells (Figure S3, Supplementary Materials), thus excluding its possible cytotoxicity. The results suggest that THQC could interrupt melanogenic signaling downstream from cAMP.

### 2.3. THQC Downregulated α-MSH-Induced Tyro Expression

**Figure 3:**
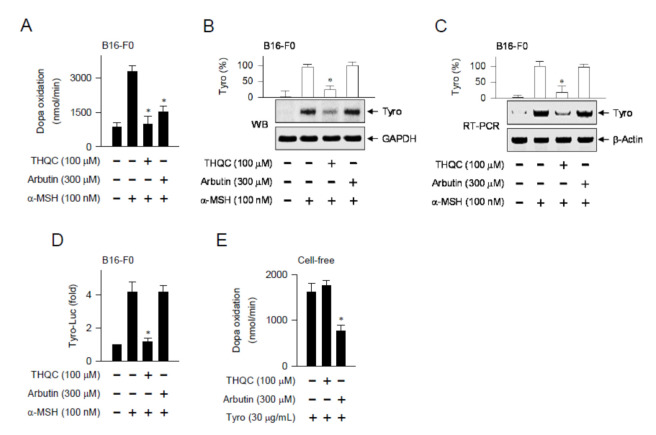
Effect of THQC on Tyro expression. B16-F0 cells were stimulated with α-MSH for 48 h (**A**,**B**) or 20 h (**C**) in the presence of THQC. (**A**) Cell extract as the enzyme source of Tyro was reacted with l-dopa as a substrate in sodium phosphate buffer, and immediately measured the increase in absorbance value at 475 nm per min. Tyro activity is represented as the initial velocity of dopa oxidation (nmol/min). (**B**) Protein extract was resolved on SDS acrylamide gel by electrophoresis and subjected to Western blot analysis (WB) with anti-Tyro or anti-GAPDH antibody. (**C**) Total RNA was subjected to semi-quantitative RT-PCR analysis of Tyro with the internal control β-actin and resolved on agarose gene by electrophoresis. (**D**) B16-F0 cells harboring the Tyro (−2236/+59)-Luc reporter in combination with the Renilla control vector were stimulated with α-MSH for 18 h in the presence of THQC. Firefly luciferase activity, reporting the promoter activity of Tyro gene, was normalized to the Renilla activity and is represented as relative fold. (**E**) Tyro protein was treated with THQC and reacted with l--dopa as a substrate in cell-free reactions. Tyro activity is represented as the initial velocity of dopa oxidation (nmol/min). * *p* < 0.05 vs. α-MSH alone (**A**–**D**) or Tyro protein alone (**E**).

We asked whether the in vivo outcomes of THQC, suppressing the expression of the *Tyro* gene in the skin, could be translated into facultative melanogenesis in B16-F0 cells. Tyro is a key enzyme in the biosynthetic pathway of melanin pigments [12]. Upon exposure to α-MSH alone, B16-F0 cells markedly enhanced the velocity of dopa oxidation, a marker of Tyro-catalyzed activity (Figure 3A). Treatment with THQC or arbutin significantly decreased α-MSH-induced Tyro activity in the cells (Figure 3A). Moreover, THQC suppressed α-MSH-induced protein levels of Tyro in B16-F0 cells, in which arbutin was not effective (Figure 3B). Moreover, THQC attenuated the mRNA levels of the *Tyro* gene in α-MSH-activated B16-F0 cells (Figure 3C).

A promoter-dependent reporter assay was then carried out using the Tyro (−2236/+59)-Luc construct. B16-F0 cells harboring the reporter construct increased luciferase activity, reporting the promoter activity of the *Tyro* gene, upon exposure to α-MSH alone (Figure 3D). Treatment with THQC inhibited α-MSH-induced promoter activity of the *Tyro* gene in the cells (Figure 3D). The results suggest that THQC could downregulate the expression of the *Tyro* gene at the transcription level. In another experiment, Tyro protein was treated with THQC or arbutin in cell-free reactions, and its catalytic activity was measured as the velocity of dopa oxidation (Figure 3E). Treatment with THQC could not directly affect the catalytic activity of Tyro protein in cell-free reactions, in which arbutin was effective as expected (Figure 3E). Thus, the antimelanogenic mechanism of THQC might be distinct from that of arbutin.

### 2.4. THQC Inhibited α-MSH-Induced Promoter Activity of the MITF-M Gene

**Figure 4:**
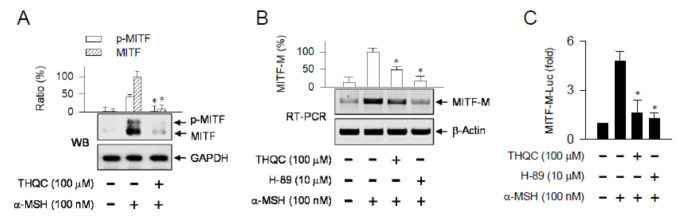
Effect of THQC on MITF-M expression. After pretreating with THQC for 2 h, B16-F0 cells were stimulated with α-MSH for 4 h (**A**) or 2 h (**B**) in the presence of THQC. (**A**) Western blot analysis (WB) of MITF with the internal control GAPDH. (**B**) Semi-quantitative RT-PCR analysis of MITF-M with the internal control β-actin. (**C**) After transfection with the MITF-M (−2200/+95)-Luc reporter in combination with the Renilla control vector, B16-F0 cells were stimulated with α-MSH for 18 h in the presence of THQC. The cell extract was subjected to a dual luciferase assay. Firefly luciferase activity, reporting the promoter activity of the *MITF-M* gene, is represented as relative fold after normalizing to Renilla activity. * *p* < 0.05 vs. α-MSH alone.

MITF-M is a master transcription factor that regulates melanogenesis in melanocytes, enhancing the transcription of *Tyro* and other melanogenic genes [9,22]. Topical treatment with THQC suppressed not only the protein levels of MITF in UVB-exposed skin (Figure 1D) but also the mRNA levels of MITF-M (Figure 1E). Similarly, α-MSH-activated B16-F0 cells markedly increased the protein levels of MITF over the basal condition (Figure 4A). Treatment with THQC suppressed α-MSH-induced protein levels of MITF in the cells (Figure 4A). Moreover, THQC attenuated the mRNA levels of MITF-M in α-MSH-activated B16-F0 cells, as did H-89 (Figure 4B). H-89 inhibits the kinase activity of protein kinase A (PKA) that is activated in a cAMP-dependent manner [28]. To understand whether THQC could affect the MITF-M promoter, B16-F0 cells were transfected with MITF-M-Luc, a reporter construct encoding the promoter region (−2200/+95) of the *MITF-M* gene. Treatment with THQC consistently inhibited α-MSH-induced promoter activity of the *MITF-M* gene in the cells, as did H-89 (Figure 4C). The results suggest that THQC could suppress the expression of the *MITF-M* gene at the promoter level. This primary action of THQC might contribute to its downstream effect that suppressed the expression of the *Tyro* gene.

### 2.5. THQC Decreased Pax3 Level for Inhibiting Facultative Melanin Pigmentation

**Figure 5:**
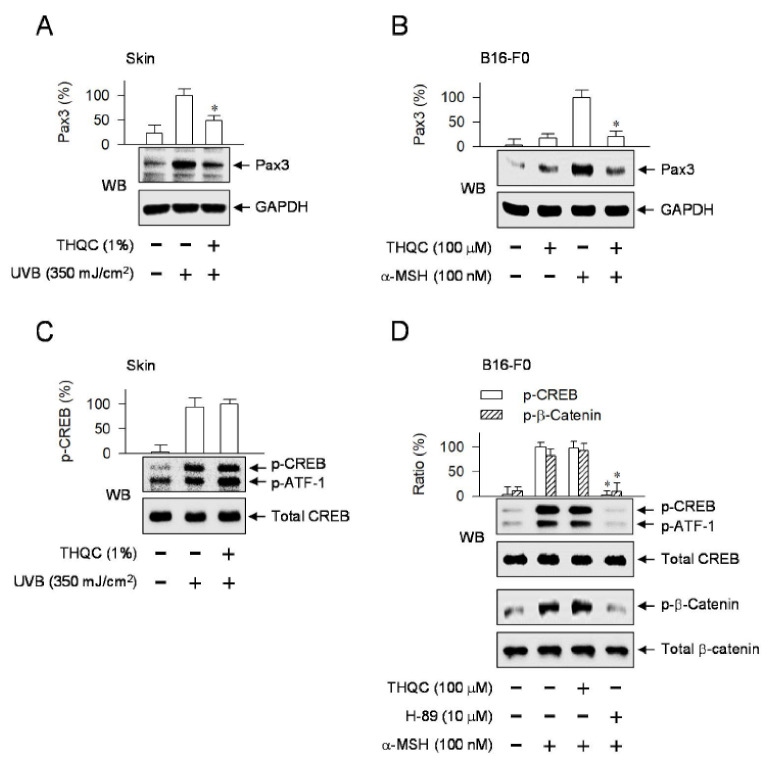
Effect of THQC on transcription factors regulating the MITF-M promoter. Transcription factors paired box gene 3 (Pax3), cAMP responsive element-binding protein (CREB), and β-catenin directly interact with the MITF-M promoter for transcriptional activation. The dorsal skin of guinea pig was irradiated with UVB and treated topically with THQC according to the protocol in Figure S1 (Supplementary Materials) (**A**,**C**). B16-F0 cells were pretreated with THQC for 2 h and stimulated with α-MSH for 2 h (**B**) or 15 min (**D**) in the presence of THQC. (**A**) Protein extract from skin tissue was resolved on SDS acrylamide gel by electrophoresis and subjected to Western blot analysis (WB) with anti-Pax3 or anti-GAPDH antibody. (**B**) Protein extract from B16-F0 culture was subjected to WB with anti-Pax3 or anti-GAPDH antibody. (**C**) Protein extract from skin tissue was subjected to WB with anti-phosphorylated (p)-CREB or anti-CREB antibody. (**D**) Protein extract from B16-F0 culture was subjected to WB with anti-p-CREB, anti-CREB, anti-p-β-catenin, or anti-β-catenin antibody. * *p* < 0.05 vs. UVB alone (**A**) or α-MSH alone (**B**,**D**).

To understand the antimelanogenic mechanism of THQC, we focused on the promoter of the *MITF-M* gene. The proximal region of MITF-M promoter encodes the *cis*-acting elements for CREB, LEF1/β-catenin, Pax3, and SOX10 that can upregulate the transcription of the *MITF-M* gene (Figure S4, Supplementary Materials). Pax3 activity on the MITF-M promoter synergizes with SOX10 in the melanogenic process [16]. The expression pattern of Pax3 protein is inducible in UVB-exposed human primary melanocytes or α-MSH-activated B16-F10 cells [29,30]. The pigmented dorsal skin of guinea pig by UVB irradiation elevated the protein levels of Pax3 over the normal skin (Figure 5A). Topical treatment with THQC significantly attenuated UVB-induced Pax3 levels in the skin (Figure 5A). Moreover, B16-F0 cells markedly increased the protein levels of Pax3 upon exposure to α-MSH alone, which was also counteracted by treatment with THQC (Figure 5B).

CREB, following phosphorylation at Ser-133, recruits CREB-binding protein (CBP) and p300 that acetylate nucleosomal histones, resulting in chromatin remodeling of target genes, including *MITF-M*, to epigenetically active [31,32]. Mitogen- and stress-activated protein kinase 1 (MSK1) directly phosphorylates CREB at Ser-133 in UVB-exposed melanocytes, in which p38 mitogen-activated protein kinase (MAPK) activates MSK1 [33]. PKA directly phosphorylates CREB at Ser-133 and β-catenin at Ser-675 in cAMP-elevated melanocytes [31,34]. The pigmented dorsal skin of guinea pig by UVB irradiation stimulated the CREB phosphorylation (p-CREB), in which anti-p-CREB antibody also cross-reacted with the phosphorylated status of activating transcription factor 1 (p-ATF-1), as shown in Figure 5C. p-ATF-1 exhibits similar structure and function to the p-CREB [35]. Topical treatment with THQC did not inhibit UVB-induced phosphorylation of CREB in the skin (Figure 5C). Upon exposure to α-MSH alone, B16-F0 cells markedly increased the phosphorylation of CREB at Ser-133, as well as that of β-catenin at Ser-675 (Figure 5D). Treatment with THQC did not affect α-MSH-induced phosphorylation of CREB or β-catenin in the cells, in which H-89 was effective as expected (Figure 5D). The phosphorylation of β-catenin protects from proteolytic degradation, accumulating in the cytoplasm, and allows it to be transported into the nucleus to function as a coactivator of LEF1 [34]. Taken together, THQC could normalize Pax3 levels in the UVB-exposed dorsal skin of guinea pig or α-MSH-activated B16-F0 culture, regulating the promoter activity of the *MITF-M* gene and resulting in antimelanogenic activity.

## 3. Discussion

Solar UVB-exposed skin stimulates melanosome biogenesis with pigmented melanin granules in melanocytes and influences the proliferation of melanocytes, which is regulated by epidermal keratinocyte- and dermal fibroblast-secreted hormones [4,36]. Excess production and aberrant distribution of melanin pigments in the skin cause hyperpigmentary disorders such as melasma, ephelides, and lentigines [37]. Melasma presents dark patches over the skin due to an increased number of melanocytes along with an enhanced capability of these cells to generate heavily pigmented melanosomes [38]. Ephelides result from excess production of melanin pigments without significant change in the number of melanocytes, while lentigines result from local proliferation of melanocytes in the skin [39]. These hyperpigmentary disorders are dermatological concerns to resolve cosmetic distress of the affected individual, demanding therapeutic strategy.

Chemical-based regulation of the pigmented disorders has been a long-standing goal for cosmetic and pharmacological applications, but their treatments remain highly challenging. Numerous screening approaches, including melanosome biogenesis and transfer in cell-based assays, have discovered hit compounds with antimelanogenic activity [40]. However, a number of the candidates limit or no success in the translation of in vitro outcomes to patients with pigmentary disorders following cutaneous application [41]. This suggests that antimelanogenic chemicals with clinical effectiveness must overcome the challenge of penetrating the skin barrier along with the stability to maintain an active configuration in skin cells. In the current study, topical treatment with THQC was protected from acquired hyperpigmentation in UVB-exposed dorsal skin of guinea pig via attenuating the mRNA and protein levels of melanogenic genes such as *MITF-M* and *Tyro*. THQC also inhibited melanin production in α-MSH-, histamine-, or db-cAMP-activated B16-F0 culture, in which THQC suppressed the expression of MITF-M at the promoter level followed by that of Tyro.

**Figure 6:**
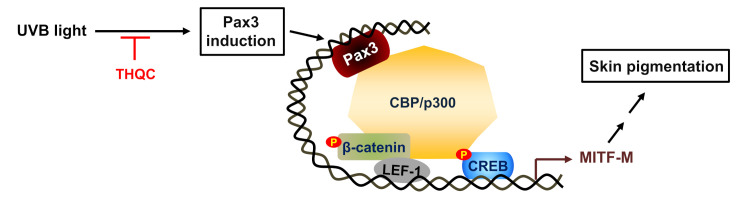
A proposed mechanism of THQC on antimelanogenic activity. Topical treatment with THQC mitigated facultative pigmentation in UVB-irradiated skin via blocking the Pax3–MITF-M axis.

We then focused on transcription factors acting on the proximal region of MITF-M promoter. Pax3 is a member of the paired box family of transcription factors, and its positive regulation of MITF-M promoter has been documented as an axis of activator protein 1 (AP-1)–transforming growth factor β (TGF-β)–Pax3 in the melanogenic process of UVB-exposed skin [29,42]. TGF-β and its signal transducers directly inhibit Pax3 expression in the skin [42]. UVB radiation represses TGF-β expression by higher activation of AP-1 in keratinocytes [29]. Low levels of TGF-β from keratinocytes inversely upregulate the expression of Pax3 in melanocytes [29,42]. In turn, Pax3 synergizes with SOX10 for transcriptional activation of the *MITF-M* gene in melanocytes [18]. UVB-exposed skin also represents cutaneous pigmentation through upregulation of the *POMC* gene [4,5]. The POMC polypeptide is then processed into α-MSH and other bioactive peptides [6]. α-MSH/MC1R signaling upregulates Pax3 expression at the transcription level in B16-F10 culture and stimulates Pax3-responsive element on the MITF-M promoter [30]. In the current study, THQC normalized Pax3 levels in UVB-exposed and pigmented dorsal skin of guinea pig, as well as those in α-MSH-activated and pigmented B16-F0 culture. However, THQC affected neither UVB-induced phosphorylation (activation) of CREB in the skin nor α-MSH-induced phosphorylation of CREB or β-catenin in B16-F0 culture. Taken together, THQC might target Pax3, a transcriptional activator of the *MITF-M* gene, without affecting other positive regulators such as CREB and LEF1/β-catenin (Figure 6).

## 4. Materials and Methods

### 4.1. Chemicals and Cell Culture

THQC was synthesized with more than 99% purity as previously described [43]. Pharmacological agents were arbutin (Sigma-Aldrich, St. Louis, MO, USA) as a skin whitener, ensulizole (Sigma-Aldrich) as a UVB absorber, and H-89 (Sigma-Aldrich) as a PKA inhibitor. Mouse melanoma cell line B16-F0 (American Type Culture Collection, Rockville, MD, USA) was cultured in Dulbecco’s modified Eagle’s medium (DMEM, Sigma-Aldrich) supplemented with 10% heat-inactivated fetal bovine serum (FBS, Corning, Manassas, VA, USA) and an antibiotic–antimycotic cocktail (GIBCO, Grand Island, NY, USA) in an atmosphere of 5% CO_2_ and 37 °C.

### 4.2. UVB-Induced Skin Pigmentation

Animal care and the experimental protocol were logged as CBNUA-1253-19-01, approved by the Animal Ethics Committee at Chungbuk National University as CBNUR-1253-19 (2019-04-04), and conducted in accordance with the Korean FDA Guide for the Care and Use of Laboratory Animals. Brownish guinea pigs (male, 6–7 weeks old) were obtained from Daehan Biolink (Eumsung, Korea). After shaving, the dorsal skin of guinea pig was allocated into six areas, irradiated with UVB light (300 mJ/cm^2^), and treated topically with THQC (0.3% or 1%), as shown in Figure S1 (Supplementary Materials). THQC was dissolved in a vehicle of propylene glycol, ethanol, and water (5:3:2). Pigmentation in the skin was measured at the end of UVB exposure using a mexameter (CK Electronic, Koln, Germany). Skin tissue was excised from the UVB-exposed area, fixed in 10% formaldehyde (Sigma-Aldrich), and embedded in paraffin (Sigma-Aldrich). Skin tissue was then sectioned at a thickness of 5 μm and stained with Fontana–Masson silver nitrate (Scytek, Logan, UT, USA). Protein extract or total RNA was prepared from UVB-irradiated skin tissue.

### 4.3. Western Blot Analysis

Cells were lysed with radioimmunoprecipitation assay (RIPA) buffer containing protease and phosphatase inhibitor cocktails (Gendepot, Barker, TX, USA) and centrifuged at 13,000 rpm for 20 min. The supernatant was collected, and its protein concentration was determined using a Bradford assay kit (Biorad, Hercules, CA, USA). Protein extract was resolved on 8–12% SDS acrylamide gel (Biosesang, Sungnam, Korea) by electrophoresis and transferred to a polyvinylidene difluoride membrane (Roche, Indianapolis, IN, USA) in a semidry condition. The blot was blocked with nonfat milk (Becton-Dickinson, Sparks, MD, USA) or bovine serum albumin (BSA, Affymetrix, Santa Clara, CA, USA) in Tris-buffered saline containing 0.05% Tween-20 (Sigma-Aldrich). After washing, the blot was incubated with primary antibody at 4 °C overnight followed by secondary antibody at room temperature for 1–3 h and reacted with an enhanced chemiluminescence (ECL) prime Western blotting detection kit (GE Healthcare, Buckinghamshire, UK). This study employed primary antibodies against Tyro (1:1000, Santa Cruz Biotechnology, Santa Cruz, CA, USA), MITF (1:1000, Abcam, Cambridge, UK), Pax3 (1:1000, Santa Cruz Biotechnology), CREB (1:1500, Cell Signaling Technology, Danvers, MA, USA), p-CREB at Ser-133 (1:1000, Cell Signaling Technology), β-catenin (1:1500, Cell Signaling Technology), p-β-catenin at Ser-675 (1:1000, Cell Signaling Technology), or GAPDH (1:2000, Santa-Cruz Biotechnology). The secondary antibody was goat anti-rabbit immunoglobulin G (IgG) labeled with horseradish peroxidase (HRP) (1:2500, Thermo Fisher Scientific, Waltham, MA, USA) or goat anti-mouse IgG labeled with HRP (1:2500, Thermo Fisher Scientific).

### 4.4. RT-PCR Analysis

Semi-quantitative RT-PCR analysis was carried out to determine the mRNA levels of Tyro or MITF-M with internal control β-actin. Total RNA was reverse-transcribed using the primer oligo-dT (iNtRON Biotechnology, Sungnam, Korea) at 42 °C for 1 h, and then subjected to PCR for 27–30 cycles using a premixed kit (Bioneer, Daejeon, Korea). Each cycle consisted of heat denaturation at 94 °C for 30 s, primer annealing at 55–60 °C for 30 s, and DNA extension at 72 °C for 1 min. Nucleotide sequences of PCR primers were as follows: Tyro (sense 5′–CATTTTTGATTTGAGTGTCT–3′, antisense 5′–TGTGGTAGTCGTCTTTGTCC–3′); MITF-M (sense 5′–TACAGTCACTACCAGGTGCAG–3′, antisense 5′–CCATCAAGCCCAAAATTTCTT–3′); β-actin (sense 5′–TGGAATCCTGTGGCATCCATGAAAC–3′, antisense 5′–TAAAACGCAGCTCAGTAACAGTCCG–3′). The RT-PCR product was resolved on agarose gel (iNtRON Biotechnology) by electrophoresis and stained with EcoDye (Biofact, Daejeon, Korea).

### 4.5. Melanin Quantification

B16-F0 cells were stimulated with α-MSH (Sigma-Aldrich), histamine (Sigma-Aldrich), or db-cAMP (Sigma-Aldrich) for 72 h. After centrifugation at 13,000 rpm for 20 min at 4 °C, the supernatant was collected as the source of extracellular melanin. After washing with medium, the cell pellet was lysed to obtain the source of intracellular melanin. Extracellular and intracellular melanin pigments were disrupted in 0.85 N NaOH and 20% dimethyl sulfoxide (DMSO, Sigma-Aldrich) by heating at 80 °C for 1 h, and then quantified by measuring the absorbance value at a wavelength of 405 nm.

### 4.6. Cell Viability Assay

A stock of 100 mM THQC was prepared by dissolution in 99% DMSO and diluted to 10 to 100 μM with medium. B16-F0 cells were incubated with THQC for 72 h in the presence of α-MSH and reacted with 0.5 mg/mL 3-(4,5-dimethylthiazole-2-yl)-2,5-diphenyltetrazolium bromide (MTT, Sigma-Aldrich) for 1 h. Formazan precipitate was dissolved in 99% DMSO and then quantified by measuring the absorbance value at a wavelength of 509 nm.

### 4.7. Dopa Oxidation Assay

Dopa oxidation velocity was measured as the catalytic activity of Tyro. The enzyme source of Tyro was reacted with 50 μM l-dopa (Sigma-Aldrich) as a substrate in 25 mM sodium phosphate buffer at 37 °C, and the increase in absorbance value at a wavelength of 475 nm per min was immediately measured for 30 min.

### 4.8. Luciferase Reporter Assay

B16-F0 cells were transfected with the reporter construct, Tyro (−2236/+59)-Luc or MITF-M (−2200/+95), in combination with the Renilla control vector using a lipofectamine kit (Invitrogen, Madison, WA, USA). The cell extract was subjected to a dual luciferase assay with a premixed kit (Promega, Carlsbad, CA, USA). Firefly luciferase activity, reporting the promoter activity of the *MITF-M* or *Tyro* gene, was normalized to the Renilla activity as a reference of transfection efficiency.

### 4.9. Statistical Analysis

Results are represented as the mean ± standard deviation from three independent experiments (*n* = 3). Data were analyzed with one-way analysis of variance (ANOVA) followed by Student’s *t*-test. A *p*-value < 0.05 was considered significant.

## 5. Conclusions

THQC mitigated melanin pigmentation in UVB-irradiated skin or α-MSH-activated B16-F0 culture via suppressing the expression of MITF-M at the promoter level, in which THQC normalized the protein levels of Pax3, a transcriptional activator of the MITF-M promoter. Taken together, we propose suppression of the axis of Pax3–MITF-M as a potential strategy in the treatment of skin pigmentary disorders that are at high risk under UVB radiation.

## Supplementary Materials

Supplementary materials can be found at https://www.mdpi.com/1422-0067/21/24/9631/s1.

## Abbreviations

CREB	cAMP responsive element-binding protein
db-cAMP	dibutyryl cyclic adenosine monophosphate
GAPDH	glyceraldehyde 3-phosphate dehydrogenase
MITF-M	microphthalmia-associated transcription factor isoform M
MSH	melanocyte-stimulating hormone
Pax3	paired box gene 3
SOX10	SRY-related HMG-box 10
THQC	tetrahydroquinoline carboxamide
Tyro	tyrosinase
UVB	ultraviolet B
