# Supplementary material for: Suppression of Pax3–MITF-M Axis Protects from UVB-Induced Skin Pigmentation by Tetrahydroquinoline Carboxamide
Source: Int J Mol Sci. 2020 Dec 17;21(24):9631. doi: 10.3390/ijms21249631 (PMC7766340; doi:10.3390/ijms21249631)
Supplement: Supplementary file 1 [file ijms-21-09631-s001.pdf]

Supplementary Materials

Suppression of Pax3-MITF-M Axis Protects from UVB-Induced Skin Pigmentation by Tetrahydroquinoline Carboxamide

Yong-Pyo Choi [1,#], Ga Hyun Kim [1,#], Song-Hee Kim [1], Jongseo Maeng [1], Heesoon Lee [1], Sang-Bae Han [1], Ki Ho Kim [2], and Youngsoo Kim [1,*]

[1] College of Pharmacy, Chungbuk National University, Cheongju 28160, Korea

[2] Kihobio Company, Cheongju 28160, Korea

[#]Y.-P.C. and G.H.K. equally contributed to this study

*Correspondence: youngsoo@chungbuk.ac.kr. Tel.: +82-43-261-2823

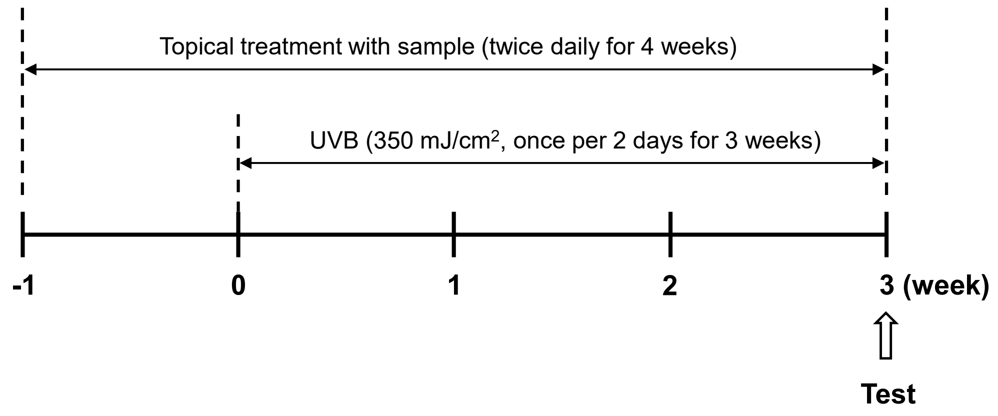

Supplemental Figure S1. Experimental protocol of skin hyperpigmentation. Dorsal skin of guinea pig was topically treated with THQC (0.3-1%) in a twice-daily regimen for four weeks, and irradiated with UV-B (350 mJ/cm^2) once every two days for three weeks. Skin hyperpigmentation was measured at the end of the UV-B exposure, and skin tissue was excised at the same time point.

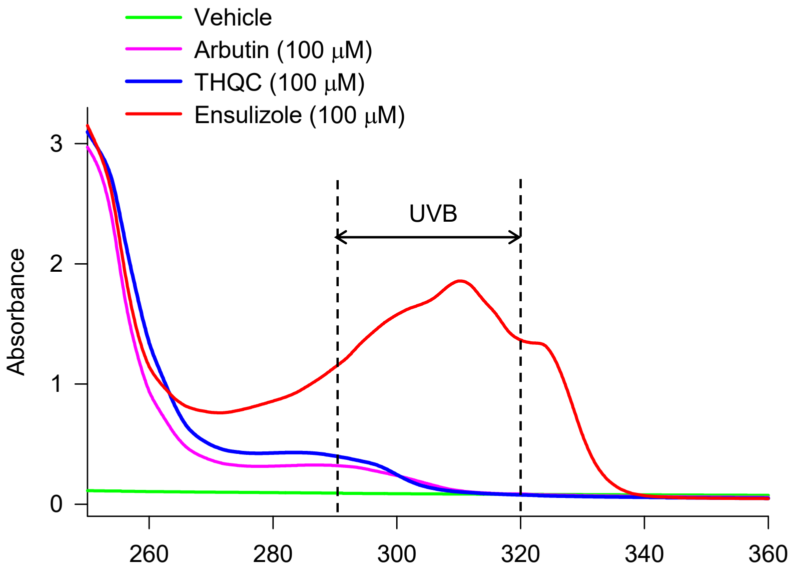

Supplemental Figure S2. Effect of THQC on UVB absorption. THQC was dissolved in a vehicle of 100% ethanol, and its absorbance values were scanned at 250-360 nm. UVB wavelength is indicated as a dotted line.

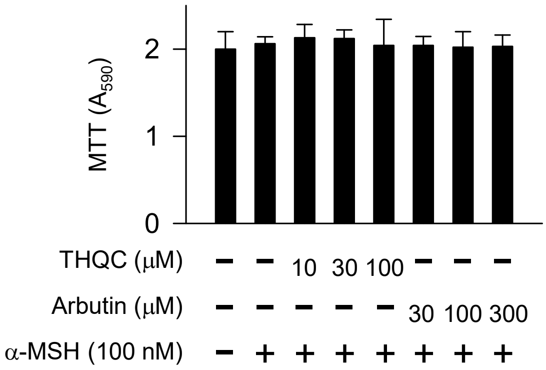

Supplemental Figure S3. Effect of THQC on cell viability. B16-F0 cells were incubated with THQC for 72 h in the presence of α-MSH, and reacted with 0.5 mg/mL MTT for 1 h. Formazan precipitate was dissolved in 99% DMSO, and quantified by measuring absorbance value at 590 nm (A_{590}).

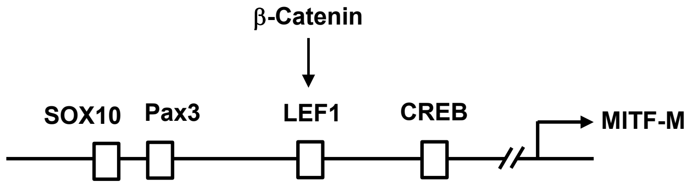

Supplemental Figure S4. Schematic representation of the proximal region of MITF-M promoter. CREB-, LEF1-, Pax3- or SOX10-responsive *cis*-acting elements on the promoter is indicated as an open box. β-Catenin assembles to LEF1 on the promoter for co-activation.
